# ROS Induced by *Streptococcus agalactiae* Activate Inflammatory Responses via the TNF-α/NF-κB Signaling Pathway in Golden Pompano *Trachinotus ovatus* (Linnaeus, 1758)

**DOI:** 10.3390/antiox11091809

**Published:** 2022-09-14

**Authors:** Jie Gao, Mingjian Liu, Huayang Guo, Kecheng Zhu, Bo Liu, Baosuo Liu, Nan Zhang, Dianchang Zhang

**Affiliations:** 1 Key Laboratory of South China Sea Fishery Resources Exploitation and Utilization, Chinese Academy of Fishery Sciences, South China Sea Fisheries Research Institute, Ministry of Agriculture and Rural Affairs, Guangzhou 510300, China; 2 Ocean College, Hebei Agricultural University, Qinhuangdao 066000, China; 3 Sanya Tropical Fisheries Research Institute, Sanya 572019, China; 4 Guangdong Provincial Engineer Technology Research Center of Marine Biological Seed Industry, Guangzhou 510300, China

**Keywords:** *Trachinotus ovatus*, pathogenic bacterial infection, histopathology, immune-related genes, inflammatory response

## Abstract

*Streptococcus agalactiae* is common pathogenic bacteria in aquaculture and can cause mass mortality after fish infection. This study aimed to investigate the effects of *S. agalactiae* infection on the immune and antioxidant regulatory mechanisms of golden pompano (*Trachinotus ovatus*). Serum and liver samples were obtained at 0, 6, 12, 24, 48, 96, and 120 h after golden pompano infection with *S. agalactiae* for enzyme activity and gene expression analyses. After infection with *S. agalactiae*, the content of reactive oxygen species (ROS) in serum was significantly increased (*p* < 0.05). Serum levels of glucose (GLU), alanine aminotransferase (ALT), aspartate aminotransferase (AST), and malondialdehyde (MDA) increased and then decreased (*p* < 0.05), reaching a maximum at 6 h. Serum antioxidant enzyme (LZM) activity increased significantly (*p* < 0.05) and reached a maximum at 120 h. In addition, the mRNA expression levels of antioxidant genes (*SOD*, *CAT*, and *GPx*) in the liver increased and then decreased, reaching the maximum at 24 h, 48 h, and 24 h, respectively. During the experimental period, the mRNA expression levels of NF-κB-related genes of the inflammatory signaling pathway inhibitory κB (*IκB*) showed an overall decreasing trend (*p* < 0.05) and the lowest expression at 120 h, whereas the mRNA expression levels of tumor necrosis factor α (*TNF-α*), interleukin-1β (*IL-1β*), IκB kinase (*IKK*), and nuclear factor NF-κB increased significantly (*p* < 0.05) and the highest expression was at 120 h. In conclusion, these results showed that *S. agalactiae* could activate internal regulatory signaling in the liver of golden pompano to induce defense and immune responses. This study is expected to lay a foundation to develop the healthy aquaculture of golden pompano and promote a more comprehensive understanding of its disease resistance mechanisms.

## 1. Introduction

Golden pompano (*Trachinotus ovatus*) is an economically important fish in China. It is mainly cultivated in deep-water cages along the southeastern coast of China. Due to its delicious meat and fast growth, it has become an essential fish in southern China [1,2]. In recent years, a large number of golden pompano have died due to *Streptococcus agalactiae* infection, causing severe economic losses to farmers [3]. There are no effective measures to prevent and treat *S. agalactiae* infection in golden pompano.

*S. agalactiae* is a Gram-positive parthenogenic anaerobic and often known as group B *Streptococcus* (GBS)*,* which can cause inflammatory disorders in animal commensals. The high pathogenicity and pathogenesis of *S. agalactiae* have been extensively studied in a range of species, including humans, rats, cows, horses, etc. [4]. It is highly pathogenic not only to terrestrial animals but also to aquatic animals. In many fish, *S. agalactiae* infection can cause multifunctional disorders and lead to high mortality. As a result, this species is of global concern as a significant pathogen in farmed fish [5]. Although scholars have reported on the pathogenesis of fish infected with *S. agalactiae*, the systematic studies of host factors in response to its infection are limited.

Serum parameters are critical indicators to measure the physiological status of the whole organism. When an organism is invaded by exogenous microorganisms, its health status can be determined by biochemical indicators in the serum [6]. Serum glucose (GLU) levels are often induced by environmental stress and are produced through glycolytic and gluconeogenic pathways to provide the energy required to combat stress [7,8]. In addition, alanine aminotransferase (ALT), aspartate aminotransferase (AST), and malondialdehyde (MDA), as sensitive indicators of the extent of oxidative damage to cells, are also commonly regulated by environmental factors [9]. Therefore, understanding changes in serum parameters is important for understanding the health status of an organism.

The invasion of pathogenic bacteria induces the production of reactive oxygen species (ROS) in the organism [2]. Excessive ROS accumulation can lead to oxidative damage to tissues and organs. As a vital antioxidant defense organ, the liver has established a complete antioxidant defense system, such as superoxide dismutase (SOD), catalase (CAT), and glutathione peroxidase (GPx) [10,11]. Therefore, the degree of oxidative damage to organisms by pathogenic bacteria can be judged by detecting the activity of antioxidant enzymes in the liver.

In addition, pathogenic infections can activate or control TNF-α/NF-κB-mediated signaling pathways [12,13]. Tumor necrosis factor α (TNF-α) and interleukin-1β (IL-1β) are the primary transcriptional regulators involved in the pathogenic invasion. Additionally, TNF-α and IL-1β function by nuclear factor-κB (NF-κB) regulatory genes through activation of classical NF-κB signaling [14]. NF-κB is a crucial regulator of cellular events and is involved in immune regulation and inflammatory and anti-apoptotic responses [15]. It is a heterodimer composed of P50 and P65 and chelated into inactive complexes under normal physiological conditions through interactions with inhibitory κB (IκB) family members [16]. When exogenous microorganisms stimulate cells, they can activate IκB kinase (IKK) through a signaling cascade [17]. IKK activates the NF-κB-IκB complex through phosphorylation of IκB and leads to ubiquitin-dependent degradation of IκB and release of NF-κB dimers from the inhibitory complex [18]. Activating NF-κB-related signaling pathways promotes nuclear translocation and transcription of NF-κB target genes, regulating proliferation, differentiation, and apoptosis or inflammation in various cell types [19,20]. In addition, IL-1β can enhance the inflammatory response by inducing NF-κB expression, creating a vicious cycle [21]. IL-1β can promote TNF-α secretion by broadly pan-activating T cells, B cells, and natural killer (NK) cells. TNF-α can promote gene transcription and exacerbate the inflammatory response by increasing the release of inflammatory factors such as IL-1β [22]. Based on the central regulation of the NF-κB pathway, the interaction of NF-κB with IL-1β and TNF-α can exacerbate inflammatory responses and lead to soft tissue contusion. Therefore, profoundly studying the immune defense mechanism of the TNF-α/NF-κB signaling pathway is necessary.

Therefore, this study assessed the effects of *S. agalactiae* infection on golden pompano by analysing serum parameters, histopathology, and the expression of genes related to the TNF-α/NF-κB signaling pathway. This study contributes to understanding the immune and defense mechanisms of *S. agalactiae* infection in golden pompano and provides a theoretical basis for its healthy breeding.

## 2. Materials and Methods

### 2.1. Ethical Statement

Animal research was approved by the Committee of the South China Sea Fisheries Research Institute, Chinese Academy of Fisheries Sciences (no. SCSFRI96-253) and performed in accordance with the applicable standards.

### 2.2. Experimental Fish and Bacteria Preparation

Golden pompano (average weight of 31.15 g) used in this experiment were healthy and energetic, obtained from the Shenzhen Experimental Base of the South China Sea Fisheries Research Institute of the Chinese Academy of Aquatic Sciences. The water temperature, salinity, dissolved oxygen, and pH range were 27 ± 2 °C, 25 ± 2%, >5.5 mg/L, and 7.8 ± 0.5, respectively. The fish were fed twice a day (9:00 am and 4:00 pm), and the feeding amount per feeding was approximately 4% of their organism mass. The strain of *S. agalactiae* was isolated from the sick golden pompano at the Shenzhen Base of the South China Sea Fisheries Research Institute in 2021. After purification and identification, it was then stored at −80 °C. The bacteria were inoculated into brain heart infusion (BHI) liquid medium and incubated at 140× *g*, 28 °C shakers for 24 h prior to the experiment. The precipitate was recovered after centrifuging the BHI liquid culture at 6200× *g* for 8 min. The pellet was then rinsed four times with sterile phosphate-buffered saline (PBS), and five concentration gradients (1.0 × 10^10^, 1.0 × 10^9^, 1.0 × 10^8^, 1.0 × 10^7^, 1.0 × 10^6^ colony-forming units [CFU]/mL) were established. A 120-h pre-experiment was then performed: one control group (PBS) and five experimental groups were included. Three replicates were set up for each group, with each replicate containing 30 fish (for a total of 540 fish). Each experimental group was injected with 200 μL of the corresponding concentration of bacterial solution for each fish, and the control group was injected with 200 μL of PBS per fish. During this period, the behavioral changes of fish were observed and recorded, and the relationship between mortality and time under different infection concentrations was studied. According to the pre-experimental results, the final 120 h half-lethal concentration (120 h LD50) of 2.0 × 10^7^ CFU/fish was obtained by multiplying the dilution of the PBS solution.

### 2.3. Experimental Infection and Sampling

Formal experiments were performed in six 150 L (140 L water) aquariums. Three hundred healthy golden pompano were selected and randomly divided equally into one control group and one infection group, each group of three parallels. Each fish in the infection group was injected with 200 μL bacteria solution at a concentration of 2.0 × 10^7^ CFU/fish. The control group was injected with 200 μL sterile PBS. Samples were collected at 0, 6, 12, 24, 48, 72, 96, and 120 h after infection. Nine fish were randomly selected at each time point in each group, and every three fish were mixed as one sample (fish were anesthetized with eugenol (40 mg/L) before sampling) [23]. The organism was sterilized with alcohol according to the previously described method [24]. Blood was collected from the caudal vein with a 1.0 mL sterile syringe and stored in a 1.5 mL centrifuge tube. After being silenced at 4 °C for 5 h, 100× *g*, 20 min centrifugation was performed to separate serum samples from blood. Serum samples from every three fish were combined into one mixed sample to obtain three, which were used for blood parameter analysis. Similarly, three fresh liver tissues were taken and immediately frozen in liquid nitrogen for enzyme activity assay and gene expression analysis. Subsequently, all samples were stored in a refrigerator at −80 °C. In addition, at 120 h, nine fish were randomly selected from the control and experimental groups, respectively. Their liver tissues were collected in sampling bottles containing 4% paraformaldehyde fixative for histological examination.

### 2.4. Histological Examination

In this experiment, liver tissue from the infection and control groups was sectioned separately by slightly modifying the protocol described by Tanaka et al. [25]. Liver tissue fixed in paraformaldehyde was first washed in 70% ethanol, dehydrated, and then embedded in paraffin using conventional techniques. Sections were cut to 5 μm, fixed on slides, and stained with hematoxylin-eosin. Images of the sections were obtained using a microscope (NIKON ECLIPSE C1) according to a previous method [26].

### 2.5. Detection of Biochemical Parameters in Serum

The contents of ROS in serum were measured using a dichloro-dihydro-fluorescein diacetate (DCFH-DA) probe according to a chemical fluorescence method. The activities of LZM, AST, and ALT were determined by the test-tube turbidimetric method. Serum levels of GLU and MDA were measured by colorimetric immunoassay and enzyme immunoassay, respectively. All the assays were performed according to the kit instructions of the Nanjing Jiancheng Bioengineering Institute (Nanjing, China).

### 2.6. Detection of Antioxidant Enzyme Activity in Liver

The activities of several important antioxidant enzymes, including SOD, CAT, and GPx, in the liver were determined using a colorimetric method according to commercial colorimetric kits (Nanjing Jiancheng Bioengineering Institute, Nanjing, China).

### 2.7. RNA Extraction and First Strand cDNA Synthesis

Total RNA was extracted from the livers of the experimental and control groups using TRIzol reagent (Cat. No. DP424, Tiangen, Beijing, China), respectively. The spectrophotometer was used to measure the absorption values of the extracted samples at 260 nm and 280 nm and an optical density (OD) ratio of 260/280. The sample 260/280 OD ratio was 1.8−2.0, indicating that the RNA concentration was in line with the requirements and could be used for subsequent experiments [27]. cDNA was synthesized according to the manufacturer’s instructions of the Prime Script™ RT reagent kit with gDNA Eraser (Accurate Biotechnology Co., Ltd., Shanghai, China). The reactions were carried out at 37 °C for 15 min and heated at 85 °C for 5 s. All cDNA samples obtained were stored at −20 °C until quantitative polymerase chain reaction (qPCR) detection.

### 2.8. Real-Time qPCR

The target gene expression was determined by qPCR using a Roche LightCycler 480 II (Roche Diagnostics, Shanghai, China). The reaction volume of qPCR was 12.5 μL. Melting curve analysis was performed based on a denaturation step at 95 °C for 30 s followed by 40 cycles at 95 °C for 5 s and 60 °C for 30 s. The experiment was repeated thrice for each sample to ensure accuracy (technical repetition) [10,28]. The primers are listed in Table 1, and the amplification efficiency was greater than 90%. *EF-1a* was chosen as the internal reference gene because it was not affected by *S. agalactiae* infection in our study. The relative expression levels of target genes relative to the control group were calculated using the 2^−ΔΔCT^ method [29].

### 2.9. Statistical Analyses

The data obtained from the experiments were analyzed by SPSS statistical software (26.0, IBM SPSS Inc., Chicago, IL, USA). All data were expressed as the means ± standard errors (SEs). Statistically significant differences between the group means were analyzed using one-way analysis of variance (ANOVA) and Tukey’s multiple comparison test, and differences were considered statistically significant at *p* < 0.05 [10].

## 3. Results

### 3.1. Toxicology of Different Concentrations of S. agalactiae on Golden Pompano

The statistical results of the death of golden pompano infected with different concentrations of *S. agalactiae* are shown in Table 2. After 120 h of infection, the survival rate of the infection group injected with doses of 2.0 × 10^9^ and 2.0 × 10^8^ CFU/fish was zero. The survival rate of the infection group with an injected dose of 2.0 × 10^7^ CFU/fish was 47 ± 3.5%, whereas no deaths were observed in the infection group with injected doses of 2.0 × 10^6^ and 2.0 × 10^5^ CFU/fish. The half-lethal time was 116 ± 5.5 h at a 2.0 × 10^7^ CFU/fish dose.

### 3.2. Examination of Liver Histopathological

**Figure 1:**
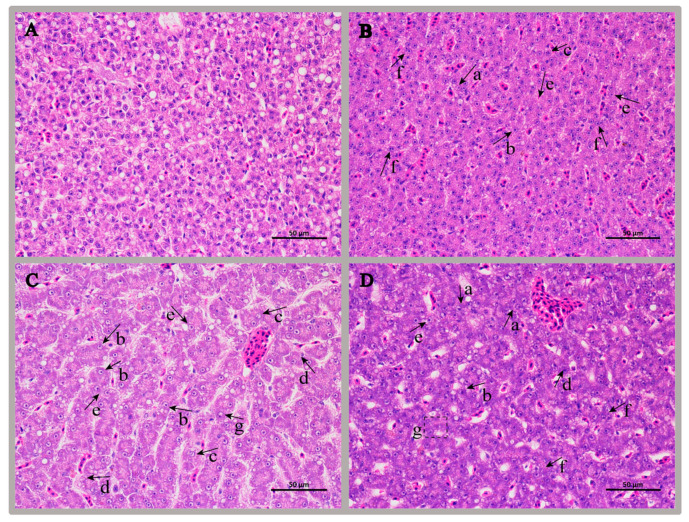
Histopathological examination of the livers of golden pompano infected with *S. agalactiae*. Notes: (**A**) represents a normal liver tissue section, HE, bar = 50 µm; (**B**–**D**) Representative liver tissue sections from three fish at 120 h of infection, respectively, HE, bar = 50 µm. a, liver sample in which liver cell boundaries were not well defined; b, liver sample with enlarged and severely displaced cells; c, liver sample exhibiting hepatic cytoplasmic lysis; d, liver sample exhibiting amyloidosis in the cells surrounding the hepatic sinusoids and cloudy eosinophilic staining of the cytoplasm; e–f, liver sample in which the cell membrane was disintegrated and the cells joined together, creating the illusion of binucleation; g, liver sample exhibiting multifocal necrosis.

At 120 h, the hepatocytes of the control group of golden pompano were slightly rounded polygonal cells. The cells were closely arranged, regular, and orderly with a clear structure, and blood cells were contained in blood vessels, as shown in Figure 1A. After 120 h of infection, the histopathology of three of the fish is shown in Figure 1B–D. Infected golden pompano liver cells were ill-defined. Their nuclei were enlarged, translucent, and severely shifted. The cells around the hepatic blood sinusoids exhibited amyloid degeneration. The eosinophilic staining of the cytoplasm was cloudy, and the cell membranes disintegrated, resulting in a double-nucleated artifact. Focal necrosis and the sporadic appearance of enlarged basophilic cells in the cytoplasm were observed in the livers of infected fish.

### 3.3. Analysis of Serum Parameters

**Figure 2:**
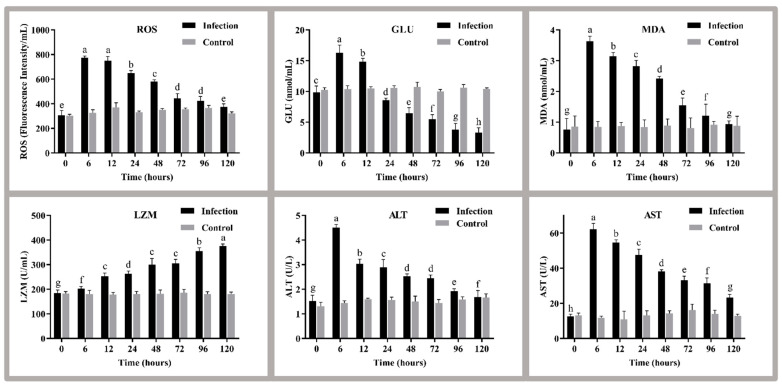
The levels of ROS, GLU, MDA, LZM, ALT, and AST in plasma at different times before and after the challenge. Different letters indicate significant differences between the stress groups (*p* < 0.05). Values are the means ± SEs (*n* = 3). The grey bars represent the indicators of fish before the challenge, and the black bars represent the indicators of fish after the challenge. ROS, reactive oxygen species; GLU, glucose; MDA; malondialdehyde, LZM, lysozyme; ALT, alanine aminotransferase; AST, aspartate aminotransferase.

The serum parameters that changed in golden pompano after *S. agalactiae* infection are shown in Figure 2. The results showed that the serum ROS, GLU, and MDA contents showed similar trends; they all trended upwards and then downwards, and they reached a maximum at 6 h after infection (772.96 fluorescence intensity/mL, 16.30 nmol/L, and 3.64 nmol/L). However, only the GLU was significantly lower than the control group after 24 h of infection (*p* < 0.05). The LZM activity gradually increased after 6 h of infection (*p* < 0.05) and peaked at 120 h (376.22 nmol/mL). ALT and AST activity tended to increase and then decrease, and they reached a maximum at 6 h after infection (4.52 U/L, 62.07 U/L).

### 3.4. Analysis of Liver Enzyme Activity

**Figure 3:**
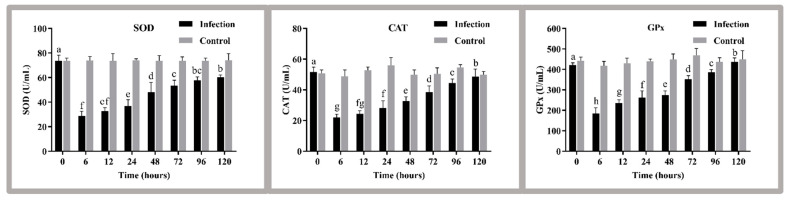
The liver antioxidant capacity of golden pompano before and after the challenge. Different letters indicate significant differences between the stress groups (*p* < 0.05). The values are the means ± SEs (*n* = 3). The grey bars represent the indicators of fish before the challenge, and the black bars represent the indicators of fish after the challenge. SOD, superoxide dismutase; CAT, catalase; GPx, glutathione peroxidase.

After *S. agalactiae* infection, the enzyme activities of SOD, CAT, and GPx in the liver all changed significantly. The activities of these enzymes decreased to the lowest level at 6 h after infection (*p* < 0.05), gradually increased, and reached normal levels (Figure 3).

### 3.5. Expression of Antioxidant Markers and Signaling Pathway Genes in the Liver

**Figure 4:**
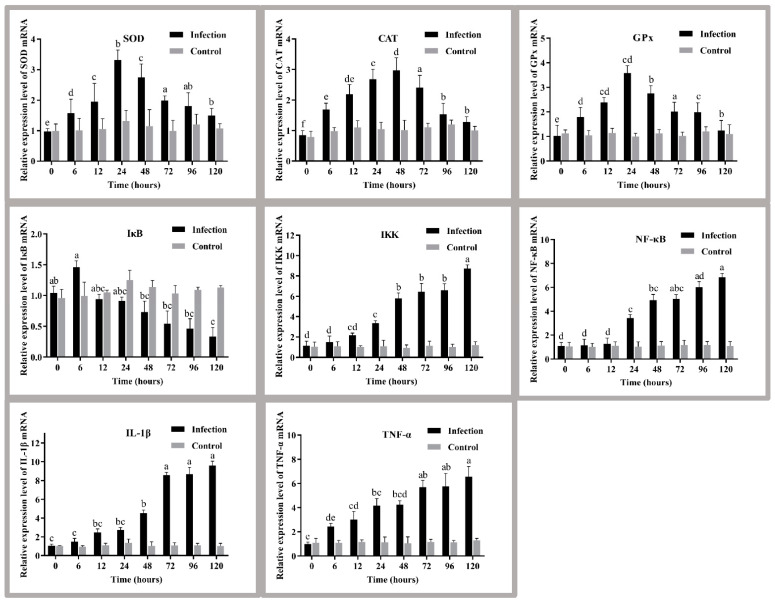
Liver antioxidant capacity and pathway genes of golden pompano before and after the challenge. Different letters indicate significant differences between the stress groups (*p* < 0.05). The values are the means ± SEs (*n* = 3). The grey bars represent the indicators of fish before the challenge, and the black bars represent indicators of fish after the challenge. IKK, inhibitor of kappa-B kinase; *IKB*, NF-kappa-B inhibitor; NF-κB, nuclear factor kappa-B; TNF-α, tumour necrosis factor α; IL-1β, interleukin 1β.

The expression levels of antioxidant genes (*SOD*, *CAT*, *GPx*) and NF-κB pathway genes (*NF-κB*, *IKK*, *IKB*) in the liver were detected by qPCR after golden pompano was infected with *S. agalactiae* (Figure 4). Specifically, the expression of the antioxidant genes *SOD*, *CAT*, and *GPx* showed a trend of first increasing and then decreasing (*p* < 0.05), reaching the maximum values at 24 h, 48 h, and 24 h, respectively. The pathway genes IKK and NF-κB were significantly upregulated at 12 h and 24 h post-infection, respectively (*p* < 0.05), whereas IκB mRNA expression showed the opposite trend. IL-1β and TNF-α mRNA expression levels gradually increased under *S. agalactiae* stimulation (*p* < 0.05), reaching the maximum at 120 h.

## 4. Discussion

In recent years, *S. agalactiae* infection has led to massive mortality of golden pompano, causing huge economic losses to the farming industry [3]. However, to date, no effective method has been found to prevent and treat this disease. Therefore, elucidating the pathological features of golden pompano after *S. agalactiae* infection from both the macro and micro perspectives is essential for its healthy farming.

### 4.1. Survival Rate and Histopathological Analysis

Survival analysis showed an inverse relationship between golden pompano survival time and *S. agalactiae* injection dose. The higher the injection dose of *S. agalactiae*, the shorter the survival time of the golden pompano. The results are consistent with studies following injection of zebrafish [33] and Nile tilapia [34] via the same pathogenic bacteria, with deaths starting at 48 h post-infection. Furthermore, previous studies have shown that *S. agalactiae* infection can invade various tissues of the host [35]. It attacks the liver tissue of the host, resulting in cell rupture and bacterial cell adhesion, and then invades the inner sinusoidal wall and the sinus cavity [35,36]. In this study, it was observed that 120 h after *S. agalactiae* infection, the cell membrane was disintegrated in the hepatocytes, and the nucleus was translucent and severely displaced. Our study showed the similar histopathological changes in hepatocytes, indicating that the model of infection with *S. agalactiae* was successfully constructed and can be used for subsequent analysis.

### 4.2. Analysis of Serum Parameter

Hematological parameters, as the main indicators of fish health status, are sensitive to bacterial infections [37]. Bacterial infections can adversely affect the oxygen-carrying capacity of the blood and the electrolyte balance of the blood, leading to extravasation of red blood cells and changes in cell size [38]. Therefore, hematological parameters are often used as important indicators to assess the health status of fish after exposure to bacterial infections and various other environmental stresses [38,39]. ROS, GLU, MDA, LZM, ALT, and AST are common serum examination parameters [39]. ROS (O, O_2_^−^, -OH, H_2_O_2_, etc.) are associated with the occurrence and development of several bacterial infectious diseases [40]. A rapid increase in ROS contents of the host in response to pathogenic bacterial stimulation can enhance the antioxidant capacity of the organism [40]. Therefore, the higher ROS contents detected in the serum of the infected group in this study compared to the control group may be due to oxidative stress caused by the invasion of *S. agalactiae* into the fish. In general, pathogenic bacteria invading the mucosal system of fish can activate LZM and trigger an immune response against pathogenic bacteria [41]. LZM activity in serum is often elevated by pathogenic bacterial stimulation [42]. Thus, the infected fish in this study showed higher LZM activity than the uninfected fish, and this activity increased over time. It is consistent with the results of LZM changes following infection of *Cherax quadricarinatus* with *Aeromonas veronii* [22]. Significant changes in GLU levels were observed in infected golden pompano, probably due to impaired hepatic glucose metabolism caused by pathogenic bacterial infection, affecting insulin resistance and glucose metabolism [43]. Serum MDA levels reflect the degree of damage after a large number of free radicals have attacked the organism. The higher its level, the greater the degree of intracellular lipid peroxidation damage [44,45]. Therefore, the persistent elevation of serum MDA levels in infected golden pompano may be related to increased lipid oxidative damage after *S. agalactiae* attack the organism. In addition, serum ALT and AST levels are also often considered important indicators for assessing the health of the liver [46]. Once liver tissue is damaged or stressed by the surrounding environment, the porosity of the plasma membrane increases, and these two enzymes in serum levels increase rapidly [47]. Therefore, this study’s elevated activities of ALT and AST may be associated with damage to hepatocytes in golden pompano.

### 4.3. Analysis of Liver Antioxidant Enzyme Parameters

Previous studies have shown that ROS do not usually cause direct damage to organisms, but rather play a role in mediating the organism’s response to various stimuli. Excess ROS can lead to an increase in free radicals (O^2−^, -OH) in the cells of the organism, which can cause oxidative damage to the organism, thereby weakening its immune defense system [22]. Fish prevent oxidative damage caused by ROS production from exposure to various environmental stress by increasing antioxidant enzyme activity. SOD can scavenge superoxide radicals (O^2−^) in organisms through disproportionation reactions, converting them to H_2_O_2_ and O_2_ [48,49]; CAT further decomposes H_2_O_2_ into H_2_O and O_2_, thereby reducing oxidative damage to the organism by free radicals [50]. In addition, the organism can be protected from ROS damage by GPx catalyzing the oxidation of GSH by H_2_O_2_ to produce oxidized glutathione (GSSG), which reduces H_2_O_2_ to non-toxic hydroxyl compounds [51]. In general, the higher the environmental stress, the greater the resistance of the organism to oxidative damage and the higher the activity of antioxidant enzymes such as SOD, CAT and GPx [52]. However, exogenous infection may also lead to a decrease in antioxidant enzyme activity due to energy expenditure during the fight against oxidative stress [53]. Therefore, an acute decrease in SOD, CAT, and GPx at the beginning of *S. agalactiae* infection in this study may be related to the rapid energy depletion of the organism at the beginning of the infection. However, with prolonged infection time, ROS accumulated excessively, and the organisms gradually increased the activities of SOD, CAT, and GPx can alleviate the damage caused by ROS.

### 4.4. Analysis of Antioxidant Markers and Signaling Pathway Genes in the Liver

This study on liver enzyme activity has confirmed that the antioxidant enzymes SOD, CAT, and GPx play an essential role in resistance to *S. agalactiae* infection. To better understand the molecular mechanisms of immunity in golden pompano against *S. agalactiae* infection, the relative expression levels of its endogenous antioxidant enzyme genes in the liver were examined first. *SOD*, *CAT*, and *GPx* gene expression levels in the liver were significantly upregulated compared to the control group. These results were similar to *SOD*, *CAT*, and *GPx* gene expression in the liver after *Aeromonas hydrophila* attacked *Channa striata* [54], suggesting that *S. agalactiae* infection affects liver antioxidant gene expression. In addition, this study indicates that the level of enzyme activity is influenced by the expression of the corresponding genes in cells. However, our study did not observe a complete agreement between the level of antioxidant enzyme activity and its gene expression level. Researchers have reported that there is no strictly linear relationship between them [55]. Therefore, further studies are needed regarding the regulatory relationship between enzyme activity and genes.

Secondly, we analysed the effect of *S. agalactiae* infection on the expression of pro-inflammatory and anti-inflammatory genes in golden pompano. It has been established that a corresponding inflammatory response is triggered when ROS accumulates to a certain level. For example, the inflammatory response activated during spring viremia of carp virus infection carp is associated with ROS accumulation [56]. As a critical transcription factor in initiating and regulating inflammation, NF-κB gene plays an essential role in the development of inflammatory response. Meanwhile, NF-κB-mediated signaling pathways are classical signaling pathways that regulate the inflammatory response [57]. This study explored the expression of NF-κB blockers and several commonly induced genes. The results showed that the expression of IKK and NF-κB increased over time in the liver of infected golden pompano, whereas the expression of IκB gradually decreased and was lower than that of the control group. It is due to pathogenic bacteria invasion resulting in the inactivation of IκB and increased NF-κB dimer activity. NF-κB dimers are activated and transferred to the nucleus through post-translational modifications to induce the expression of multiple genes, producing multiple cytokines associated with inflammation [58,59]. Furthermore, NF-κB, a central regulator of the inflammatory response, plays a central role in inducting and encoding pro-inflammatory cytokines IL-1β and TNF-α [59,60]. Thus, IL-1β and TNF-α, as stress-inducible genes, are consistently upregulated in expression during infection [58].

## 5. Conclusions

In this study, serum biochemical indices, histopathology, and expression of TNF-α/NF-κB pathway genes after infection with *S. agalactiae* were investigated using golden pompano as experimental subjects. This study shows that serum biochemicals could be used to indicate the healthy status of golden pompano after infection with *S. agalactiae*. TNF-α/NF-κB has an essential immunomodulatory role in the resistance to *S. agalactiae* infection in golden pompano. In conclusion, our results may provide a theoretical basis for disease prevention and treatment of golden pompano.

## Figures and Tables

**Table 1:** Primers used for amplification and mRNA expression analysis.

Primer Name	Primer Sequences (5′−3′)	Amplification Target	Reference
*SOD*-F	CCTCATCCCCCTGCTTGGTA	qPCR	[2]
*SOD*-R	CCAGGGAGGGATGAGAGGTG		
*CAT*-F	GGATGGACAGCCTTCAAGTTCTCG	qPCR	[2]
*CAT*-R	TGGACCGTTACAACAGTGCAGATG		
*GPx*-F	GCTGAGAGGCTGGTGCAAGTG	qPCR	[2]
*GPx*-R	TTCAAGCGTTACAGCAGGAGGTTC		
*IKK*-F	CCTGGAGAACTGCTGTGGAATGAG	qPCR	[30]
*IKK*-R	ATGGAGGTAGGTCAGAGCCGAAG		
*IκB*-F	GCTGGTCCATTGCCTCCTGAAC	qPCR	[30]
*IκB*-R	GTGCCGTCTTCTCGTACAACTGG		
*NF-κB*-F	TGCGACAAAGTCCAGAAAGAT	qPCR	[31]
*NF-κB*-R	CTGAGGGTGGTAGGTGAAGGG		
*IL-1β*-F	CGGACTCGAACGTGGTCACATTC	qPCR	[32]
*IL-1β*-R	AATATGGAAGGCAACCGTGCTCAG		
*TNF-α*-F	GCTCCTCACCCACACCATCA	qPCR	[10]
*TNF-α*-R	CCAAAGTAGACCTGCCCAGACT		
*EF-1α*-F	AAGCCAGGTATGGTTGTCAACTTT	qPCR	[10]
*EF-1α*-R	CGTGGTGCATCTCCACAGACT		

**Table 2:** Survival rate of golden pompano induced by intraperitoneal injection of *S. agalactiae* and the survival time corresponding to each treatment.

Dosage (CFU/fish)	Survival Rate at 120 h (%)	Half-Lethal Time (LT_50_, h)	Survival Time (h)
2.0 × 10^9^	0	17 ± 1.0	16~19
2.0 × 10^8^	0	37 ± 3.0	36~70
2.0 × 10^7^	47 ± 3.5	116 ± 5.5	95~125
2.0 × 10^6^	100	130 ± 2.5	130~150
2.0 × 10^5^	100	>160	>160

## Data Availability

Data are contained within the article.
